# Pharmacological Inhibition of Caspase and Calpain Proteases: A Novel Strategy to Enhance the Homing Responses of Cord Blood HSPCs during Expansion

**DOI:** 10.1371/journal.pone.0029383

**Published:** 2012-01-03

**Authors:** Sangeetha V. M., Darshana Kadekar, Vaijayanti P. Kale, Lalita S. Limaye

**Affiliations:** Stem Cell Biology Laboratory, National Centre for Cell Science, Ganeshkhind, Pune University Campus, Pune, Maharashtra, India; Ohio State University Medical Center, United States of America

## Abstract

**Background:**

Expansion of hematopoietic stem/progenitor cells (HSPCs) is a well-known strategy employed to facilitate the transplantation outcome. We have previously shown that the prevention of apoptosis by the inhibition of cysteine proteases, caspase and calpain played an important role in the expansion and engraftment of cord blood (CB) derived HSPCs. We hypothesize that these protease inhibitors might have maneuvered the adhesive and migratory properties of the cells rendering them to be retained in the bone marrow for sustained engraftment. The current study was aimed to investigate the mechanism of the homing responses of CB cells during expansion.

**Methodology/Principal Findings:**

CB derived CD34^+^ cells were expanded using a combination of growth factors with and without Caspase inhibitor -zVADfmk or Calpain 1 inhibitor- zLLYfmk. The cells were analyzed for the expression of homing-related molecules. *In vitro* adhesive/migratory interactions and actin polymerization dynamics of HSPCs were assessed. *In vivo* homing assays were carried out in NOD/SCID mice to corroborate these observations. We observed that the presence of zVADfmk or zLLYfmk (inhibitors) caused the functional up regulation of CXCR4, integrins, and adhesion molecules, reflecting in a higher migration and adhesive interactions *in vitro*. The enhanced actin polymerization and the RhoGTPase protein expression complemented these observations. Furthermore, *in vivo* experiments showed a significantly enhanced homing to the bone marrow of NOD/SCID mice.

**Conclusion/Significance:**

Our present study reveals another novel aspect of the regulation of caspase and calpain proteases in the biology of HSPCs. The priming of the homing responses of the inhibitor-cultured HSPCs compared to the cytokine-graft suggests that the modulation of these proteases may help in overcoming the major homing defects prevalent in the expansion cultures thereby facilitating the manipulation of cells for transplant procedures.

## Introduction

Expansion of stem/progenitor cells from cord blood is an important area of research in the field of clinical/experimental hematology. Despite the recent advancements in this field, investigations have shown that the expanded grafts exhibit altered biological functions. Among the cellular changes, the important functional impairment seen is the reduced/defective homing ability of these cells in culture thereby hampering their transplantation potential. Homing is an important prerequisite for engraftment, which utilizes the ability of HSPCs to migrate, adhere and lodge within the bone marrow niches and involve the synergistic action of adhesion molecules and integrins [1]–[3]. Previous studies revealed that the defective homing observed in cultured cells is due to the down regulation of beta integrins and chemokine receptors especially CXCR4, which is an indispensible homing molecule crucial for the trafficking of stem cells into the bone marrow following intravenous infusion [4], [5]. CXCR4 is further implicated in the maintenance of HSC quiescence, therefore the apparent down regulation may affect the HSC maintenance as well as engraftment [6], [7].Though a plethora of contributing factors have been under scrutiny, the exact molecular mechanism behind these altered features is still elusive.

The role of cell cycle, survival cues and that of integrin signaling in altering the homing properties has been evaluated in earlier studies [8], [9]. Interestingly, the role of apoptosis in causing a homing defect in the cultured HSPCs was also documented [10], [11]. Moreover, it was shown that 24 hours of cytokine treatment is enough to predispose the HSPCs to apoptosis, reducing their engraftment ability thereby linking apoptosis as a factor that affects the cellular functions [12]. Previously, we identified a distinctive aspect of apoptosis (negative regulation) that influenced the expansion of CD34^+^ cells. Our study revealed that the cell-permeable inhibitors of caspase and calpain proteases, augmented the expansion and long-term engraftment of cord blood CD34^+^ cells [13]. We speculated that the mechanism behind the sustained engraftment might lie in the ability of the inhibitors to influence the migratory and adhesive interactions of these cells *in vitro.*

To explore this aspect, we studied the homing responses of cytokine-expanded HSPCs (Control) with those that were additionally supplemented with the inhibitors, zVADfmk/zLLYfmk. In agreement with our hypothesis, we noticed that the presence of protease inhibitors in a cytokine-containing culture system positively influenced the cell polarization, migratory and adhesive responses and *in vivo* homing of expanded cells. The current results together with our previous observations emphasize the importance of the modulation of these proteases for generating functionally superior HSPCs in an *ex vivo* system.

## Results

### HSPCs expanded in the presence of zVADfmk or zLLYfmk showed a higher CXCR4 expression

**Figure 1:**
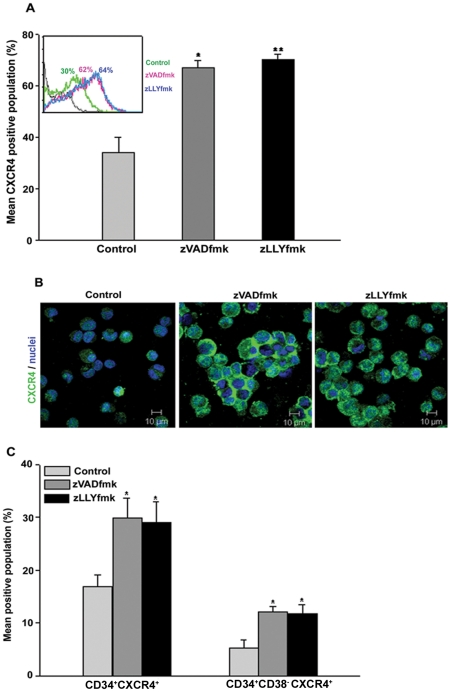
CXCR4 expression analysis of the cultured HSPCs. (A) The expanded cells were stained using anti CXCR4 antibody and the expression was assessed by flow cytometry. HSPCs cultured in the presence of zVADfmk/zLLYfmk showed a two-fold increase in the CXCR4^+^ population compared to the control counterpart. Data are represented as mean percentage ± standard deviation of five biological replicates, *p≤0.05, **p≤0.01. A representative flow-overlay is shown in the inset. (B) Confocal microscopy images further confirm higher expression of CXCR4 (green) on cell surface of HSPCs from zVADfmk/zLLYfmk cultures than that of controls. Cell nuclei (blue) were stained with DAPI (bar  = 10 µm, n = 3). (C) Flow cytometry analyses of HSPCs after multicolor staining (CD34 and CXCR4 or CD34, CD38 and CXCR4) demonstrate higher CXCR4 positive population in CD34^+^ and CD34^+^CD38^−^ compartments. Thus, zVADfmk/zLLYfmk facilitates higher production of CXCR4 expressing CD34^+^ and CD34^+^CD38^−^ hematopoietic progenitor cells. Data are represented as mean percentage ± standard deviation of four experiments, *p≤0.5.

CXCR4-SDF1 interaction is crucial for stem cell homing and retention. Since CXCR4 expression is reportedly altered during expansion, we analysed the expression of CXCR4 protein in our optimized culture conditions. The expansion of CB-derived HSPCs in the presence of zVADfmk/zLLYfmk resulted in a two-fold increase in CXCR4 positive population (**Figure 1A**, *p≤0.05, **p≤0.01, n = 5). A representative flow cytometry profile is shown in inset (**Figure 1A**). Percent CXCR4 population ranged from 30%±3.35, 60%±2.54 and 61%±3.89 in the control, zVADfmk and zLLYfmk sets respectively. Images captured under confocal microscope after immunostaining of the expanded cells also showed a higher expression of CXCR4 on the HSPCs cultured in the presence of inhibitors (zVADfmk/zLLYfmk) compared to the control (**Figure 1B**). Since the CD34^+^ cells expressing CXCR4 are important for HSC functions, the expanded cells were further assessed for the co-expression of these two markers. It was found that the sets containing zVADfmk/zLLYfmk, retained a two-fold higher number of CD34^+^CXCR4^+^ population compared to the control. On an average CD34^+^CXCR4^+^ subsets ranged from 15.85%±2.32 in control compared with the 29.85%±3.87 in zVADfmk and 29.05%±3.9 in zLLYfmk containing cultures. Moreover, we also observed an increase in the proportion of cells expressing CD34^+^CD38^−^CXCR4^+^ phenotype (5.2%±1.64, 12.067%±0.984 and 11.73%±1.76 in control, zVADfmk and zLLYfmk respectively) indicating the retention of primitive progenitors to a higher extent in the cultures (**Figure 1C**, * *p*≤0.05, n = 4, **Figure S2A and S2B**).

### Migration towards Stromal Derived Factor 1-α (SDF1) was enhanced in the zVADfmk/zLLYfmk HSPCs

**Figure 2:**
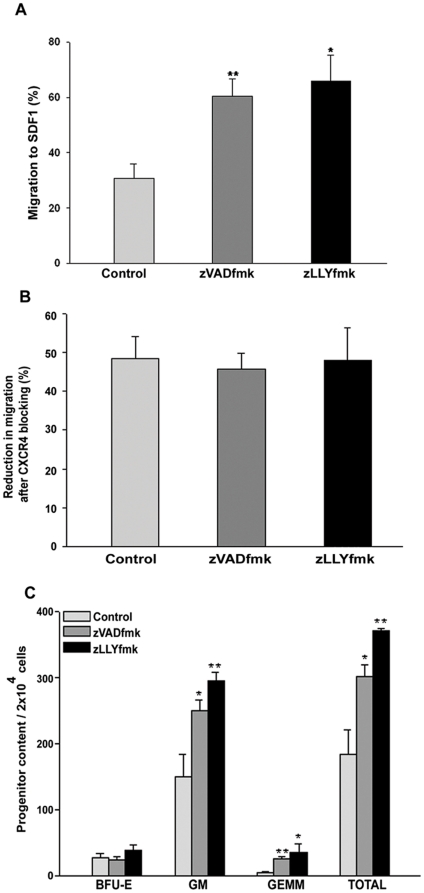
*In vitro* migration of expanded CB HSPCs. (A) The presence of zVADfmk/zLLYfmk during *ex vivo* expansion significantly increased the chemotaxis of HSPCs towards SDF1α compared to the cytokine-expanded (control) counterpart. Data are represented as mean ± standard deviation of four experiments, *p≤0.05, **p≤0.01. (B) The migration of expanded HSPCs towards SDF1α was reduced when a neutralizing antibody to CXCR4 was used, indicating the direct role of CXCR4 in maintaining the chemotactic responses. Data are represented as mean ± standard deviation of three experiments. (C) The colony forming assay of the migrated population showed a higher clonogenicity, especially the presence of primitive progenitors in terms of CFU mix/GEMM colonies in the zVADfmk/zLLYfmk cultures. Data obtained from three experiments are represented as mean ± standard deviation, *p≤0.05, **p<0.01.

Since the CXCR4 (putative receptor of chemokine SDF1α), expression was up regulated; we next compared the chemotactic responses of the control and zVADfmk/zLLYfmk HSPCs towards SDF1α. We observed about a two- fold increase in the migration ability of the HSPCs that were cultured with inhibitors (60%) as compared to the control (30%) (**Figure 2A**, *p≤0.05, **p≤0.01, n = 4). Thus the data demonstrated that the higher CXCR4 expression in zVADfmk/zLLYfmk HSPCs was functional in maintaining stronger chemotactic responses. We also observed that the colony forming ability of migrated fraction of the zVADfmk/zLLYfmk HSPCs were superior to that of control cells when subjected to methyl cellulose based colony assays (**Figure 2C**, *p≤0.05, **p<0.01, n = 3). The number of Burst forming unit erythroid (BFU-E) ranged from 28.66±5.5, 25.33±5.03 and 39±7.8 in control, zVADfmk and zLLYfmk respectively. The Granulocye Macrophage (GM) colonies were significantly increased in zVADfmk 250±15.04 and in zLLYfmk (295.6±11.71) compared to control (150±34.69) . There was also an increase in the CFU GEMM (Granulocyte - Erythrocyte - Macrophages and Megakaryocytes) colonies in the migrated fraction of inhibitor cultured HSPCs (4.66±2.88 in control vs 26.0±3.0 and 36.33±12.055 in zVADfmk and zLLYfmk) relative to the control (4.66±2.88).

As a next step, we sought to confirm the specificity of CXCR4 mediated migration. Neutralizing antibodies for blocking the surface CXCR4 on HSPCs was employed prior to migration. A significant reduction in migration was observed in all the three sets after CXCR4 blocking (**Figure 2B**, n = 3). Since expanded cells are more heterogeneous in nature and our previous results showed that zVADfmk/zLLYfmk expands the functional CD34^+^ cells, we further examined whether treatment with these inhibitors afforded a ‘priming’ effect on the immature CD34^+^ cells.

To study this aspect, the isolated CD34^+^ cells were incubated overnight with cytokines in presence or absence of the protease inhibitors and were then subjected to chemotaxis towards SDF1α. We noticed an enhanced migration of CD34^+^ cells, incubated with zVADfmk/zLLYfmk compared to the control **(Figure S2C**, **p<0.01, n = 4). The percent migration was 57±7.5, 82.25±7.3 and 76.25±6.2 in controls, zVADfmk and zLLYfmk respectively. Interestingly, though there was improved migration in the test sets, the CD34^+^ cell number was found to be unaltered as assessed by flow cytometry (>90%, as seen from **Figure S2D**). We further observed that this increase in migration owed to a higher CXCR4 expression levels in these sets. The CXCR4 population ranged from 11.0%±0.913 in control, 17.750%±1.708 in zVADfmk and 18.50%±2.887 in zLLYfmk after overnight incubation. A representative flow cytometry profile is shown in **Figure S2E**, n = 4). Furthermore, phenotypic analyses of the migrated fraction revealed that there was higher percentage of CD34^+^CD38^−^ subset in the treated set, suggesting the positive influence of these inhibitors in ‘priming’ the homing responses of immature CD34^+^ cells (**Figure S2D**, n = 4).

### Presence of zVADfmk/zLLYfmk during expansion enhanced the expression profile of adhesion molecules on the cultured HSPCs

**Figure 3:**
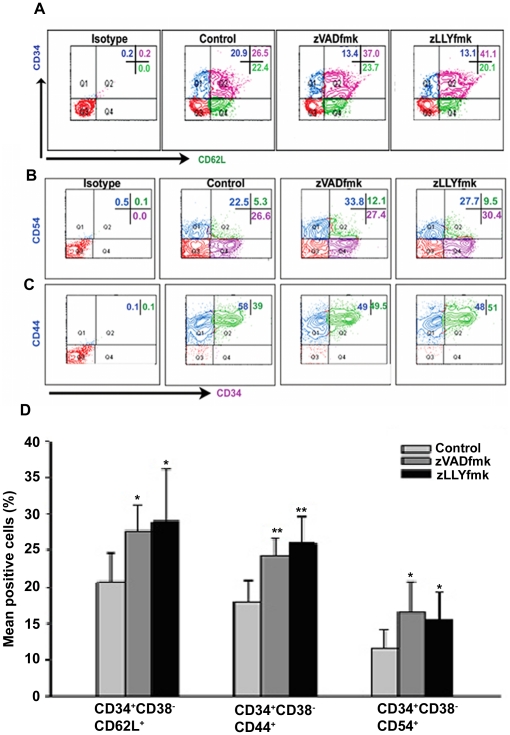
Higher expression of adhesion molecules in the expanded HSPCs. (A) & (B & C) Flow cytometry analyses of HSPCs after staining for CD34 vs CD62L, CD34 vs CD54 and CD34 vs CD44 antibodies demonstrate higher positive population of these adhesion molecules on CD34^+^ progenitor cells. (D) Alternatively multi colour flow cytometry analyses showed that zVADfmk/zLLYfmk enhanced the presence of CD62L, CD44 and CD54 on CD34^+^CD38− subsets as well. Data are represented as mean percentage ± standard deviation of four experiments, *p<0.05, **p<0.01.

The adhesion of HSPCs to endothelial wall/ECM proteins is mediated by the synergistic action of selectins, cell adhesion molecules (CAMs) and integrins. Flow cytometry analysis showed a higher percentage of CD62L positive cells co-expressing CD34 marker in the inhibitor cultured HSPCs after expansion (**Figure 3A**). We also observed that the total expanded HSPCs from zVADfmk/zLLYfmk sets retained a higher percentage of CD62L (L selectin) population (44.75%±8.05 in zVADfmk and 43.75%±5.6 in zLLYfmk) relative to the control cells (27.25%±2.2) (**Figure S3 A**, *p<0.05, n = 6). Confocal imaging showed that these cells also stained intensely for CD62L, compared to the control (**Figure S3B**). While speculating a logical reason behind this up regulation, we analyzed the expression level of CD62L cleaving enzyme TACE (ADAM-17) after expansion. Interestingly, we observed that the presence of zVADfmk/zLLYfmk resulted in a simultaneous reduction in the cells expressing TACE, which is a known sheddase of CD62L. The TACE positive population ranged from 48%±4.5 in control compared to the 32.3%±5.3 in zVADfmk and 31.5%±4.8 in zLLYfmk HSPCs (* *p*≤0.05, n = 4). A representative flow profile from an experiment is shown in **Figure S3C**. The results indicated that the inhibitors of caspase and calpain significantly lowered the percentage of cells expressing TACE, pointing out to the possibility of retention of CD62L upon higher survival cues. Apart from selectins, the profile of adhesion molecules including CD54 (Intercellular Cell Adhesion molecule-1/ICAM-1) and CD44 (Hyaluronan Cell Adhesion molecule/HCAM) was also found to be influenced in these culture conditions. A higher number of CD34^+^ cells in zVADfmk/zLLYfmk cultures co-expressed CD54 and CD44 on their surface. CD34^+^CD54^+^ cells ranged from 6.61%±2.02 in control, 11.8%±1.0 in zVADfmk and 10.73%±1.09 in zLLYfmk respectively. The cells co-expressing CD34 and CD44 ranged from 34%±5.5 in control vs 44.6%±4.4 and 48.3%±3.05 in zVADfmk and zLLYfmk respectively (n = 3). Representative flow cytometry dot plot is depicted in **Figure 3B and 3C**. We also checked whether the presence of zVADfmk/zLLYfmk enhanced the adhesion molecule profile on primitive cells within the expanded compartment by carrying out multi color staining on expanded cells. As shown in **Figure 3D**, the inhibitor treated HSPCs retained a higher percentage of CD62L, CD44 and CD54 on CD34^+^CD38^−^ fraction of expanded cells compared to the control (* *p*≤0.05, ** *p*≤0.01, n = 3). Immunostaining of the cells also showed a higher protein density of these two CAMs on the cell surface of zVADfmk/zLLYfmk HSPCs compared to the control (**Figure S3D**, upper and lower panels n = 3).

### zVADfmk/zLLYfmk enhanced the expression of integrins CD49d/VLA-4 and CD49e/VLA-5, potentiating the adhesive interactions to fibronectin

**Figure 4:**
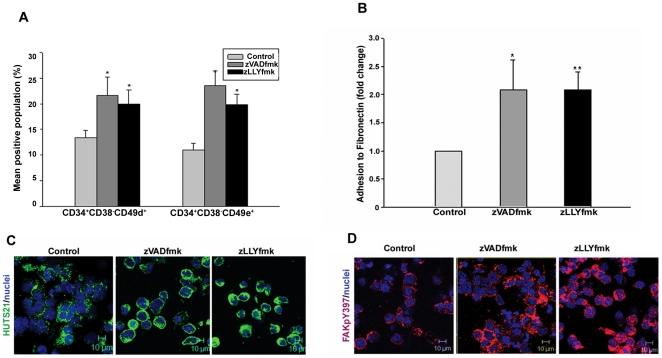
Improved expression of CD49d and CD49e integrins and higher adhesion of the expanded HSPCS. (A) Multicolour flow cytometry analysis showed a an increase of CD49d and Cd49e integrins in the primitive CD34^+^CD38^−^ fraction of inhibitor-treated HSPCs. Data are represented as mean percentage ± standard deviation of four experiments, *p<0.05. (B) Adhesion to extracellular matrix protein fibronectin was significantly increased (up to two fold) in the zVADfmk/zLLyfmk cultures compared to the control. Data are represented as mean ± standard deviation of four experiments, *p<0.05. (C) The adhered zVADfmk/zLLYfmk HSPCs retained a higher β1 integrin ligand binding status and the activation of focal adhesion kinase (D) compared to the control, green- HUTS 21, red – FAKpY397, blue – nuclei, scale 10 µm.

The integrins CD49d and CD49e are important mediators in the homing and engraftment of HSPCs. Given the improved profile of adhesion molecules, we were curious to analyse whether these caspase and calpain inhibitors affected the presence of major integrins (CD49d/VLA-4 and CD49e/VLA5) on these cells. Multicolor imunostaining followed by flow cytometry analysis showed that zVADfmk/zLLYfmk HSPCs increased the percentage of integrins on CD34^+^CD38^−^ subset of the expanded pool (**Figure 4A**, *p<0.05, n = 3). CD34^+^CD38^−^ CD49d^+^ and CD34^+^CD38^−^ Cd49e^+^ in control was 13%±1.52 and 11%±1.32. In zVADfmk the expression was 21.66%±3.15 and 23.5%±3.85 respectively. zLLYfmk showed 20.0%±2.64 and 19.83%±2.02 percentage of the primitive subset respectively. However, we did not observe any significant change in the percentage of cells expressing these integrins on total expanded cells. The percentage of cells expressing CD49d and CD49e in control was 85%±5.0 and 77.66%±2.517. In zVADfmk the prescentage was 89%±3.606 and 81.66%±7.638, and in zLLYfmk the same was 90%±2.646 and 83%±4.58 respectively. In the meantime, we noticed that the inhibitor – cultured cells showed a higher mean fluorescence intensity of these integrins relative to the control as assessed from flow cytometry **(Figure S4A**, *p<0.05, **p<0.01, n = 4). Since integrin functions are heavily dependent upon the affinity status rather than the expression (inside-out signaling), we proceeded to map the functional activity/affinity epitope of the β1 integrins using HUTS21 mAb. We observed that the zVADfmk/zLLYfmk HSPCs exhibited a higher expression of HUTS 21 compared to the control pointing out to a functionally active conformation of integrins on them (**Figure S4B**). When subjected for adherence on ECM protein fibronectin, these cells showed a two-fold increase in the adhesion assessed from the cell-matrix adhesion assay (**Figure 4B**, *p<0.05, n = 4). Since fibronectin mediated adhesion activates the integrins and forms firm focal adhesions, we again assessed the activity-epitope of integrins in the HSPCs that were adhered to the fibronectin. As seen from **Figure 4C**, the adhered HSPCs in the zVADfmk/zLLYfmk cultures exhibited its active ligand binding status compared to the control cells. Furthermore, these adhered cells showed an enhanced activation of Focal adhesion kinase as detected using FAKpY397 immunostaining (**Figure 4D**).

### Effect of blocking of integrins and adhesion molecules on the *in vitro* migration and adhesion of expanded HSPCs

**Figure 5:**
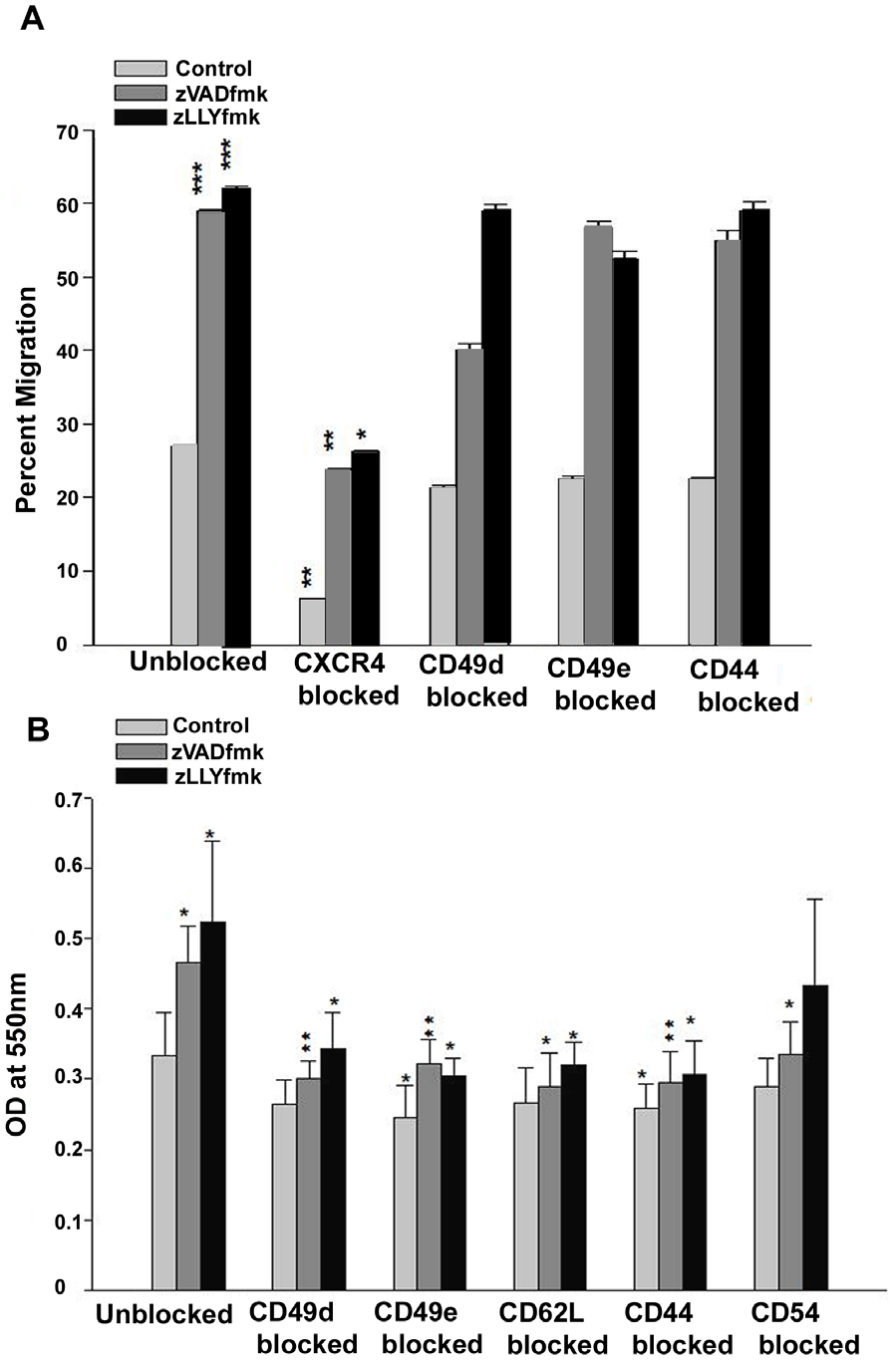
Effect of blocking of integrins and adhesion molecules on the migratory and adhesive properties. (A) *In vitro* migration of expanded control and inhibitor-treated HSPCs to SDF1 was not affected by blocking of the integrins alone. Data are represented as mean ± standard deviation of three experiments, *p<0.05, **p<0.01, ***p<0.01. (B) However, the blocking of integrins and adhesion molecules reduced the *in vitro* adhesive interaction of expanded HSPCs to a higher extent. Data are represented as mean ± standard deviation of three experiments, *p<0.05, **p<0.01.

The enhancement in the integrin and adhesion molecule profile after expansion was an important observation. We wanted to investigate these differences with respect to the functional significance of these molecules in the expanded cells. To explore this part,*in vitro* migration to SDF1α and adhesion to ECM protein fibronectin was carried out after a series of function blocking experiments for CXCR4, CD49d, CD49e, CD44, CD62L and CD54. We noticed that the trans-well chemotaxis of expanded control and inhibitor treated HSPCs significantly reduced when CXCR4 was blocked (as in accordance with the previous observation in **Figure 2**). Neither the blocking of CD49d and CD49e showed an effect in the trans-well assay, nor the adhesion molecules, implying the dominance of CXCR4-SDF1 axis (**Figure 5A** *p<0.05, **p<0.05, n = 3). However, neutralizing the integrins (CD49d & CD49e) and cell adhesion molecules significantly reduced the adhesive interactions to fibronectin, even though the effect of CD54 blocking was moderate (**Figure 5B**, *p<0.05, **p<0.05, n = 3).

### Caspase and calpain inhibitors, positively modulated the actin polymerization and enhanced the expression of RhoGTPase members: RhoA, Rac1 and Cdc42

**Figure 6:**
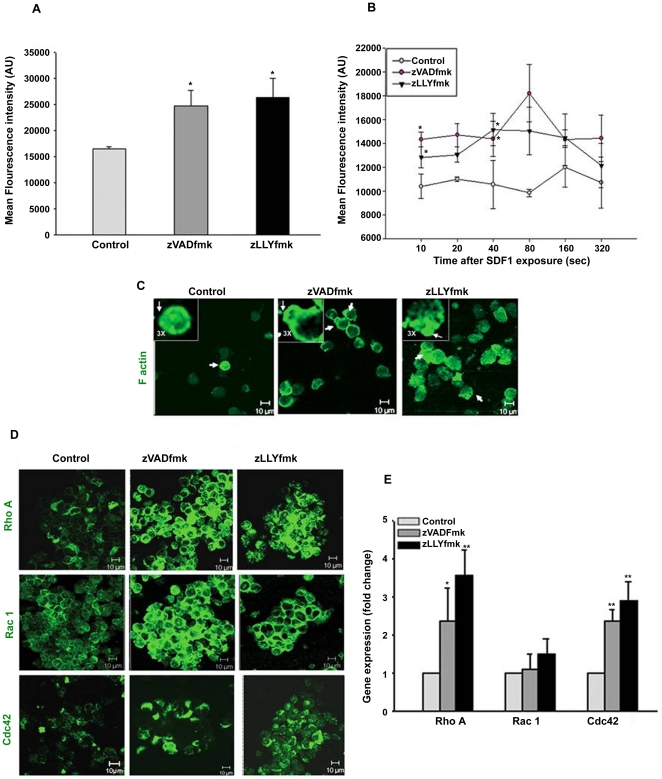
Actin dynamics of expanded HSPCs. (A) The expanded control and zVADfmk/zLLYfmk HSPCs were assessed for the polymerized actin (F actin) using fluorescein conjugated phalloidin. The presence of zVADfmk and zLLYfmk enhanced the of F actin fluorescence intensity compared to the control. Data are represented as mean ± standard deviation of four experiments, *p≤0.05. (B) Actin polymerization assay done by exposing the control and test HSPCs to SDF1α for different time intervals showed an early response and higher polymerization of actin at all time points analyzed in the zVADfmk/zLLYfmk sets. Data are represented as mean ± standard deviation of three experiments. Statistical analysis was always made between control vs. zVADfmk/zLLYfmk at all time points. *p≤0.05, **p<0.01. (C) The migrated population from the zVADfmk/zLLYfmk HSPCs showed a typical polarized morphology with a prominent localization of F actin towards the leading edge of the cells (white arrow heads & insets) where as in control cells a homogeneous distribution of F actin was seen without much visible cell polarization, green – F actin, bar = 10 µm. (D) The expression of RhoGTPase members RhoA, Rac1 and Cdc42 was found to be increased in the zVADfmk/zLLYfmk HSPCs, green –RhoA, Rac1 and Cdc42, bar = 10 µm (E) Quantitative real time PCR analysis showing a significant up regulation in the gene expression of RhoA upto (3.2 fold) and Cdc42 (up to 2.5 fold) in the zVADfmk/zLLYfmk expanded HSPCs compared to the control, whereas the Rac1 showed a modest increase in those sets. Data are represented as mean ± standard deviation of three experiments, *p≤0.05, **p<0.01.

Cytoskeletal re-arrangements play an important role in the adhesive and migratory interactions in various cell systems. To explore this aspect in the expanded HSPCs, the polymerized actin content of the control and zVADfmk/zLLYfmk sets were analyzed separately after the 10^th^ day of culture. We found that the mean intensity (MFI) of the fluorescein-conjugated phalloidin was significantly higher in the test sets as quantified from flow cytometry. The MFI ranged from 16409±443 in control vs 24719±685 in zVADfmk and 26334±1210 in zLLYfmk respectively (**Figure 6A**, *p≤0.05, n = 4). We then investigated the extent and kinetics of actin polymerization in the cultured HSPCs by exposing the cells to chemokine SDF1α for different time periods. It was observed that at all time points analyzed; the mean intensity of F actin was markedly higher in the zVADfmk/zLLYfmk sets compared to the control. The kinetics revealed a rapid increase in the actin polymerization at 40 sec after the SDF1 exposure in the test sets, which further peaked at 80 sec especially in the zVADfmk HSPCs. In the mean time, the control cells showed a delay in F actin formation and the extent of polymerization peaked only after 160 sec of chemokine exposure. This indicated that the observed advantage of the zVADFmk/zLLYfmk HSPCs in the adhesion and migration may be associated with a higher and early response to the chemokine mediated F actin formation (**Figure 6B**, *p<0.05, n = 3). In parallel experiments, the F actin distribution was visualized in the HSPCs migrated to SDF1 gradient by confocal microscopy. As shown in **Figure 6C**, Filamentous actin showed a homogenous distribution in the migrated control HSPCs, where as the zVADfmk/zLLYfmk HSPCs showed a notably more polarized distribution of F actin, towards the leading edge of the cells (**Figure 6C**, inset, a 3× magnified area) indicating a higher cell polarization. Given the importance of RhoGTPase members in actin remodeling and polymerization, our next step was to analyze the status of Rho GTPase members after the *ex vivo* expansion. Immunostaining and subsequent confocal imaging of Rho family members, RhoA, Rac1 and Cdc42 revealed an enhanced expression of these proteins in the zVADfmk/zLLYfmk HSPCs (**Figure 6D**, n = 3). Rac1 transcripts showed a modest increase compared to the control. However, there was around 3.2 fold and 2.5 fold increase in the RhoA (a transcipt abundance of 1× 10^7^±0.1×10^7^ in control vs 3.2×10^7^±0.2×10^7^ in zVADfmk and 3.1×10^7^±0.3×10^7^ in zLLYfmk) and Cdc42 transcript abundance (1.3×10^7^±0.1×10^7^ in control vs 3.2×10^7^±0.1×10^7^ in zVADfmk and 3.6×10^7^±0.5×10^7^ in zLLYfmk respectively assessed by real time PCR. These observations indicate a positive influence of caspase and calpain inhibition on active actin remodeling (**Figure 6E**, *p<0.05, **p<0.01, n = 3).

### *In vivo* homing of zVADfmk/zLLYfmk HSPCs was superior to that of the cytokine cultured cells and was mediated through the CXCR4 axis and integrins

**Figure 7:**
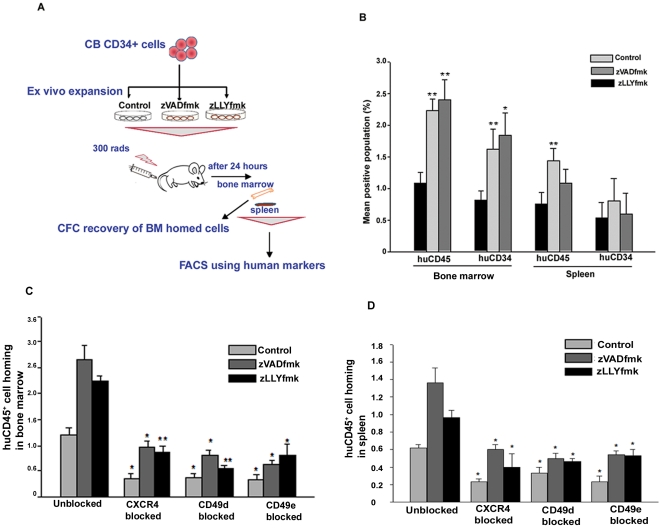
*In vivo* homing expanded HSPCs. (A) Schematic illustration of the *in vivo* homing assay conducted in NOD/SCID mice. (B) After 24 hours of transplantation, the control and test animals were sacrificed and the bone marrow and spleen cells were assessed for the presence of human CD45 and human CD34 markers. The animals that received the HSPCs expanded in the presence of either of the inhibitors exhibited a significantly increased migration to the bone marrow and spleen tissues. Data are represented as mean ± standard deviation of four experiments, *p≤0.05, **p<0.01. However, the CD34^+^ cell homing to the spleen tissues were found to be unaffected. (C & D) Blocking of CXCR4 and integrins prior to transplantation significantly reduced the bone marrow and spleen homing of expanded HSPCs compared to the unblocked respective sets. The zVADfmk/zLLYfmk HSPCs showed an increase in homing compared to control (*p≤0.05, **p<0.01, n = 3 independent experiments).

Our previous studies showed that the zVADfmk/zLLYfmk HSPCs were superior in forming a sustained long-term multilineage engraftment in NOD/SCID mice. The observations so far showed superior homing properties of HSPCs expanded in the presence of caspase/calpain inhibitors. To validate our current *in vitro* observations, we carried out an *in vivo* short-term homing experiments in the NOD/SCID mouse model as illustrated in **Figure 7A**. Equal numbers of expanded control and zVADfmk/zLLYfmk cells were infused intravenously into sub-lethally irradiated NOD/SCID mice. After 24 hours of transplantation, the bone marrow and spleen cells were immunostained for human specific CD45 and CD34 antibodies as described in methods. Flow cytometry analyses showed a recovery of significantly higher number of huCD45^+^ cells from the bone marrow of animals that received zVADfmk/zLLYfmk HSPCs. The percentage of huCD45^+^ cells in the bone marrow ranged from 1.08%±0.174 in control animals compared to 2.24%±0.175 and 2.4%±0.324 in animals that received inhibitor cultured HSPCs (**Figure 7B**, **p≤0.01, n = 4 independent experiments). To analyze the donor CD34^+^ progenitor homing, the recovered marrow cells were also stained using human specific CD34. We further observed a higher number of human CD34^+^ cells in the bone marrow of the irradiated recipient that received the zVADfmk/zLLYfmk HSPCs relative to the control. The range of huCD34^+^ progenitors recovered was 0.82%±0.146 in control, 1.62%±0.322 in zVADfmk and 1.84%±0.36 in zLLYfmk respectively (**Figure 7B**, *p≤0.05, **p<0.01 n = 5 independent experiments). Similarly the presence of donor CD45^+^ cells in the spleen of the animals that received zVADfmk/zLLYfmk HSPCs were also higher. However, we noticed that the homing of CD34^+^ progenitors to the spleen was low and was unaffected by the presence of inhibitors. (huCD45^+^ and huCD34^+^ cells in spleen ranged from 0.76%±0.181 and 0.54%±0.234 in control compared to 1.44%±1.93 and 0.8%±0.36 in zVADfmk and 1.08%±2.3 and 0.6%±0.32 in zLLYfmk) Representative flow cytometry profiles are shown in **Figure S5A–S5B**. The CFC recovery of the human cells from the bone marrow was significantly higher in the animals those received zVADfmk/zLLYfmk cultures confirming the above observations (5.2±3.4, in control to that of 16.3±5.1 and 11.5±1.5 in zVADfmk and zLLYfmk, **Figure S5C**, *p<0.05, n = 3). These observations thus clearly indicated that, the presence of caspase and calpain inhibitors augmented the *in vitro* homing responses of the expanded CB cells thereby exhibiting a higher and directed homing *in vivo*, paving an ideal situation for sustained engraftment.

To further assess the effect of the CXCR4-SDF1 axis, integrins and adhesion molecules in short-term homing ability of expanded HSPCs *in vivo*, we used blocking antibodies for the above markers to block surface receptors prior to homing assay. After 24 hours of transplantation, the bone marrow and spleen cells were analysed for the presence of human cell population by staining using antihumanCD45 antibody. We observed that blocking of CXCR4 and integrins had a profound negative effect in the homing of HSPCs to the bone marrow and spleen of irradiated recipient compared to the respective unblocked sets (**Figure 7C–7D**, *p<0.05, *p<0.05, n = 3 independent experiments). The inhibitor expanded cells indeed showed an increase in homing compared to control in these conditions. However, we did not observe any significant reduction in the homing responses of HSPCs to bone marrow and spleen when blocking antibodies for CD44, CD62L and CD54 was employed. (**Figure S6A–S6B**).

## Discussion

Expansion of the hematopoietic stem/progenitor cells (HSPCs) are attempted worldwide to increase the number of HSCs and HPCs for transplantation purposes. The feasibility of this method has been demonstrated [14]–[16] but the major impediment observed was the apparent change in the functions of the cultured HSPCs. The biological or physiological changes occurring to these cells *in vitro*, result in an ‘altered behavior’ explained by the loss of ‘stemness’, increased susceptibility towards apoptosis, defective homing and engraftment [17]. Earlier study by Traycoff et al showed that the proliferation-induced decline of primitive HSPC activity was coupled with an increase in apoptosis in the expanded CD34^+^ cells [18]. Of note various investigators have shown a possible role of apoptosis cascade in the regulation of stem cell numbers [19]–[21]. Our belief regarding the role of apoptotic proteases was rooted in our previous observation showing the effect of caspase inhibitor; zVADfmk and calpian1 inhibitor; zLLYfmk in expansion protocols (in the enhancement of CD34^+^ cell content and multilineage engraftment of expanded HSPCs).

In the present study, we demonstrate another unique aspect of this inhibition in boosting the *in vitro* and *in vivo* homing functions of CB HSPCs. Our observation of functional up regulation of CXCR4, upon protease inhibition followed by enhanced migration was an important one, as several studies have shown engraftment defects owing to reduced CXCR4 functions [6], [22]. The presence of higher number of multipotent progenitors (GEMM) colonies in the migrated fraction of zVADfmk/zLLYfmk HSPCs indicated an enrichment of primitive progenitors. To exclude the possibility that the observed differences in the migration are not merely due to the differences in the progenitor content after culture, we checked the effect of short-term incubation of these inhibitors on isolated CD34^+^ cells. We observed an enhancement in the migration of primitive CD34^+^CD38^−^ in the zVADfmk/zLLYfmk “primed” CD34^+^ cells compared to the cytokine exposed counterpart. This observation was interesting and set forth the notion that the inhibition of these proteases may have a broader implication than expected. Moreover, it was proved from the flow data that this enhanced migratory effect was prominent on the long term engrafting CD34^+^CD38^−^ subset. It would be noteworthy to mention that we also observed a general reduction in the migration efficiency of control cells (from 60% after short-term incubation to 30% after 10^th^ day of culture) compared to the zVADfmk/zLLYfmk HSPCs (75% vs. 60% respectively) clearly pointing out to the functional impairment of cytokine-cultured cells during *ex vivo* expansion.

The mechanisms of homing have revealed a host of contributing factors including integrins and selectins, CD44, complement proteins, certain lipid mediators, and intracellular signaling molecules [23]–[25]. Our observation of higher numbers of CD34^+^ CD62L^+^ cells after expansion was crucial as these populations are reportedly important predictors of the transplant outcome [26]. Surprisingly, we also noticed a lower percentage of HSPCs expressing ADAM-17/TACE (CD62L cleaving enzyme) in the zVADfmk/zLLYfmk sets. Various reports supported this notion that TACE activity can be induced upon cellular apoptosis through intrinsic and extrinsic pathways followed by CD62L shedding [27], [28]. It can also be contemplated that the reduced apoptosis levels in the inhibitor expanded cells might have helped in higher CD62L retention on them. However, the exact specificity of TACE needs to be further verified to make a conclusive statement. We did observe a reduction in the adhesion of HSPCs *in vitro* when a neutralizing antibody to CD62L was used. However, the blocking of CD62L was found to have no effect on trans-well migration of expanded cells to SDF1 α. In the *in vivo* scenario as well, homing to bone marrow was least affected by the blocking of CD62L, whereas a moderate reduction was seen in spleen homing. The role of HCAM1/CD44 is undoubtedly established in the homing of HSPCs as several groups have shown the interaction of CD44 and lodgment of HSCs in the BM [29], [30]. Whether the higher CD44 protein density on the inhibitor expanded HSPCs correlate with a higher fucosylation/glycation status is an aspect that require further studies as this mechanism was found to be important for its functionality [31]. Similar to the above observation, blocking of CD44 was also not sufficient to reduce the bone marrow homing, (correlating with the *in vitro* migration after CD44 blocking) even though it did reduce *in vitro* adhesion of HSPCs. Here we cannot exclude the possibility of not properly spanning the functional epitopes of CD44/CD62L as glycation/sialyl groups are indispensable for the functional activation of CD44/CD62L [32].

Besides CD44 and CD54, we also observed a higher expression of vinculin (data not shown) which is known to play a prominent role in the anchoring of F actin to the membrane. Recently an indispensable role of vinculin in HSPCs engraftment has also been reported opening up the multifaceted functions of adhesion molecules in HSC biology [33]. The reduced adhesion receptor expression and functional binding of integrins to matrix proteins was also discussed as the molecular basis of defective homing [34]. Moreover, a recent study documented the negative aspect of calpain proteases in focal adhesion dynamics and migration [35].

We show that the presence of caspase/calpain inhibitors positively influence the functional conformation of β1 integrins without affecting the percentage of cells in total HSPCs expressing them (inside-out signaling) thereby augmenting the adhesive interactions to the ECM protein fibronectin. Since focal adhesions are the integrin-mediated contacts between the cytoskeleton and ECM, the integrin activation induces FAK to autophosphorylate on Tyr-397. We observed a similar scenario on the adhered zVADfmk/zLLYfmk HSPCs as they showed a higher expression of pFAKy397 indicative of the activated state of focal adhesion kinases usually observed when it is bound to β1 integrins. We observed that the neutralizing antibodies to CD49d and Cd49e significantly reduced the adhesion of HSPCs to fibronectin *in vitro.* Interestingly, we did not observe any added effect of combined blocking of integrins on *in vitro* adhesion (data not shown). Surprisingly the trans-well migration to SDF1 *in vitro* was also not affected unless the integrins were blocked together with CXCR4 (data not shown). This clearly indicated that the observation of reduced migration was solely attributed to the blockade of CXCR4. This may also be explained by the lack of a trans-endothelial barrier in our *in vitro* experiments that can transduce integrin-mediated signaling for migration. We presume that in such a situation the, CXCR4-SDF1 axis gains dominance independent of integrins to induce migratory responses *in vitro.*

Prior to migration, cells display a complex repertoire of motility-associated processes, including shape changes, polarization, actin polymerization, and polarized adhesion [36], [37]. It has been reported that chemokine-induced induction of actin polymerization is most pronounced within the first 1 to 2 minutes after stimulation of the receptor and is also superior in CB HSCs compared to the PB derived HSCs. We observed a consistent polymerization of cortical actin till 160 sec post SDF1α exposure in accordance with the published reports [38], [39]. However, comparing the control and treated HSPCs, we infer that the cytokine culture alone (control) delayed the cell polarization and motility of HSPCs in response to chemokine signaling, adding to another reason for their reduced migration. We reasoned that enhanced F-actin polymerization in the expanded CB cells may owe to the differences in the expression of signaling components leading to RhoGTPase activation. The higher expression of RhoA, Rac1 and Cdc42 in the zVADfmk/zLLYfmk HSPCs indicated the possibility of involvement of these components as well. RhoA, Rac1 and Cdc42 are found to act synergistically to enhance the actin polymerization and homing interactions in HSPCs, even though diverse functions are assigned to them in various systems [40], [41]. Higher Cdc42 expression is also implicated in HSC self-renewal and quiescence as the studies employing conditional knock down of Cdc42 in murine model showed defects in maintaining HSC quiescence and retention in the bone marrow [42].

Attempts to enhance the homing and engraftment of transplanted cells are in the forefront of investigations mainly because not all HSPCs home efficiently. Studies including small molecule agonists, prostaglandin E2, and the inhibition of dipeptidyl peptidase IV (CD26) have been reported to enhance the homing of both murine and human HSCs [43]–[45]. The fact of impaired homing exhibited by cytokine-activated cells in NOD/SCID mice was previously reported [10], [11]. Other studies also demonstrated that the homing of the HSPCs was followed by a phase of extensive apoptosis and the homed cells survived and expanded only when they reach BM [46]. However, several contradictory reports exist regarding the proliferation of cells after immediate transplantation/homing [47]–[49]. Since our major aim being the comparison of homing efficiency of cytokine cultured vs. zVADfmk/zLLYfmk cells, we did not address this particular aspect in the current study.

Nonetheless, the immunostaining of recovered cells from the recipient mice using human CD45 and CD34 markers revealed that the presence of zVADfmk/zLLYfmk during expansion significantly enhanced trafficking of HSPCs to the bone marrow and spleen compared to the control. These results implied an ideal scenario for sustained engraftment. Our *in vivo* homing assay further showed that the enhanced homing of zVADfmk/zLLYfmk HSPCs was importantly mediated through CXCR4-integrin axis. We noticed that the bone marrow and spleen homing of expanded HSPCs was significantly reduced when blocking antibodies for either CXCR4 or integrins (CD49d & CD49e) were used. The blocking of CD44, CD62L and CD54 did not affect the BM homing, though a mild reduction was seen in spleen homing that lacked significance to assign an importance. The differences in the observation revealed by the blocking of adhesion molecules in an *in vitro* cell-ECM adhesion system and *in vivo* homing may be explained by the fact that “homing” involves adhesion as well as migration of which latter is more important for BM lodgment. This is also supported by the fact that homing and engraftment is dependent on a threshold level of functional integrins and not merely on the adhesion molecule expression. However, a comparison of the effect of blocking of the above factors on freshly isolated vs expanded HSPCs will further strengthen our understanding on any differential effect of the adhesion molecules in these two experimental conditions.

Nonetheless, through this study, we demonstrate a hitherto unknown aspect of the regulation of apoptotic proteases in augmenting the adhesive and migratory interactions of cultured HSPCs through up regulation of functional integrins and CXCR4. Thus the mechanism of the augmented *in vivo* homing by zVADfmk/zLLYfmk may be partly mediated through the CXCR4-SDF1 and integrin axis. The study further suggests that the use of zVADfmk/zLLYfmk in combination with cytokines can be envisioned as a promising approach to enhance the homing potential of the HSPCs. The outcome of this study may also complement the existing protocols for expansion and transplantation of stem/progenitor cells.

## Methods

### Collection of Umbilical cord blood and isolation of CD34^+^ cells

Cord blood samples were obtained after full-term deliveries and collected from local hospitals with written informed consent of the mother. The institutional review board committee named Institutional ethical committee, National Centre for Cell Science (IEC, NCCS) specifically approved this study. From cord blood, mononuclear cells were separated using ficoll hypaque (density 1.077 g/ml, Sigma Aldrich, St.Louis MO) density gradient centrifugation. CD34^+^ cells were isolated from the mononuclear cells using Dynal beads as per manufacturer's instructions (Dynabeads M-450 CD34; Dynal, ASA, Oslo, Norway).

### Expansion of CD34^+^ cells in serum free medium

Isolated CD34^+^ cells were seeded at a density of 5×10^4^cells per 500 µl of Stempro medium (GIBCO, Grand Island, NY), in 24-well tissue culture plates (Falcon, Beckton Dickinson, San Jose, CA). Growth factors used were IL-6, SCF, TPO, and Flt-3-L (Peprotech Inc, Rocky Hill, NJ) at a final concentration of 25 ng/ml with (test cells) and without (control cells) the addition of either zVADfmk −100 nM or zLLYfmk −10 µM (MP Biomedicals, Aurora, Ohio). After the 10^th^ day of culture, the cells were collected from the suspension culture, and centrifuged (1000 rpm for 5 minutes). The cells were used for assessing the *in vitro* homing properties or were used for *in vivo* homing studies in NOD/SCID mice.

### Flow cytometry analysis

HSPCs were analyzed for the expression of CXCR4, CD34, CD38, CD62L/L-selectin, CD49d/VLA-4, CD49e/VLA-5, CD54, CD44 (all antibodies from BD pharmingen- San Jose, California) and ADAM-17/TACE (R&D systems, Minneapolis, USA). The expanded HSPCs were collected, washed and suspended in 1× phosphate buffer saline (PBS) containing 0.1% bovine serum albumin (BSA). After the addition of antibodies, the cells were incubated for 40 min on ice, washed using 1× PBS and were acquired on FACS. Appropriate isotype controls were kept for each set. Total cells in the Forward Scatter (FSC) and Side Scatter (SSC) pattern were gated after excluding the debris. A minimum of 10,000 events for each sample was acquired on FACS CantoII (Beckon Dickinson San Jose California) and analyzed using FACS Diva software (Beckon Dickinson San Jose California).

### Immunofluorescence and Confocal microscopy

The expression of CXCR4, CD44, CD54, HUTS 21, FAKpY397 (BD Pharmingen, San Jose, California), RhoA, Rac1 and Cdc42 (Santacruz biotech, Santacruz, CA, USA) were analyzed. All primary antibodies were used at 1∶50 dilutions. Goat anti mouse monoclonal IgG FITC (BD pharmingen, San Jose, California) and donkey anti mouse Cy3 (Chemicon Internationals Billerica, MA 01821 USA) were used at 1∶100 dilution. For staining, the cells were fixed in 4% paraformaldehyde, permeabilized using chilled 50% methanol (as per requirement) and blocked with PBS containing 1% BSA for 1 hour. For visualizing the F actin by phalloidin staining, the cells were permeabilized using 0.1% Triton X 100. Cells were incubated overnight at 4°C with primary antibodies, washed with PBS and then incubated with the respective secondary antibodies along with DAPI (1 µg) at room temperature for 1 hour. Slides were washed three times using 1× PBS and mounted using mounting medium. For staining of the cells adhered to fibronectin, adhesion assay was carried out in chamber slides (Thermo Fischer) and the adhered cells were fixed in 4% paraformaldehyde followed by immunostaining as described above. Confocal images were obtained using a Zeiss LSM 510 Meta laser-scanning microscope. Results presented are representative fields confirmed from a minimum of three different biological samples.

### RNA isolation and Real-time PCR analysis

Total RNA was isolated using Trizol reagent (Sigma Aldrich, St Loius MO) as per manufacturer's instructions. After extraction the quantitation of RNA was done using nanodrop spectrophotometer (ND1000). A ‘high capacity cDNA archive kit’ (Applied Biosystems, Foster City, CA) was employed to carry out the first strand cDNA synthesis using random hexamers. 1 µg RNA of each sample was reverse transcribed to cDNA in 10 µl of final volume. The real time PCR was performed using the Power SYBR-Green master mix (Applied Biosystems, Foster City, CA) and specific gene primers in a 7500 ABI-prism sequence detection system (Applied Biosystems). The final reaction volume was 20 µl for each gene and the thermal condition used was 50°C for 2 min, 95°C for 10 min, (95°C for 15 sec, 60°C for 1 min) for 40 cycles. Samples without reverse transcription were used as negative controls. All qRT-PCR values were normalized to 18s and calculated according to the manufacturer's instructions. The primer sequences used are listed in **Figure S1**.

### Trans-well migration assay

Chemotactic responses of the expanded control and test HSPCs to stromal derived factor 1 (SDF1α) were assessed *in vitro* by trans-well migration assay. Briefly, 10^5^ of expanded cells were seeded into the upper chamber of the 8 µ insert. 100 ng of SDF1α was added to the lower wells containing 600 µlof medium. For each experimental set, some wells were kept without SDF1α to detect the spontaneous migration. The cells were allowed to migrate for 5 hours at 37°C. The migrated cells were collected, manually counted and subjected to colony formation assays. To check the effect of these inhibitors on CD34^+^ cells per se, freshly isolated CD34^+^ cells were incubated overnight with growth factors in the presence or absence of zVADfmk/zLLYfmk. The CD34^+^ cells were then collected washed to remove the medium and was analyzed for CD34, CD38 and CXCR4. The cells were then subjected to migration experiment as described above. In parallel experiments, the migratory responses of expanded cells were studied after blocking of CXCR4, CD49d, CD49e, CD44, CD62L and CD54 receptors employing neutralizing antibodies.

### Colony Forming Unit assay

The expanded HSPCs those migrated to SDF1α were subjected to clonogenic assays as described [50]. After 14 days of culture, colony forming unit of Granulocytes-Macrophages (CFU-GM), Burst Forming Unit Erythroids (BFU-E), and mixed colonies of Granulocyte- Erythroid-Macrophage- Megakaryocytes (GEMM) were scored under an inverted microscope. Colony assay of NOD/SCID BM cells were also carried out to detect the human CFC recovery. The mouse bone marrow cells (2×10^6^) were kept for adherence (1 hr, 37°C, 5% CO_2_) to remove the stromal cells and the non – adherent fraction was collected (approximately 10^6^ cells) and subjected to CFU assay using human specific growth factors and the colonies were scored after 2 weeks.

### Cell – matrix adhesion assay

Adhesion of the expanded HSPCs to fibronectin was carried out as per Miao et al [51]. Briefly 10^5^ each of expanded control and test HSPCs were seeded on fibronectin-coated plates which were previously blocked using 1% BSA (37°C, 1 hour) to reduce the non – specific binding. The plates were incubated for 35 minutes (37°C, 5% CO_2_). The loosely adhered cells were gently washed off and the adhered cells were fixed using 4% paraformaldehyde (10 min, room temperature) and then stained using crystal violet stain (0.1% in 70% methanol). The plates were washed three times using sterile distilled water to remove the excess stain and were then permeabilized using 0.1% Triton X 100 for 30 minutes, at room temperature. The optical density (OD at 550 nm) was read using an ELISA reader. The higher OD corresponded to a higher adhesion. In parallel experiments the adhesion of expanded HSPCs were studied after blocking of CD49d, CD49e, CD44, CD62L and CD54 receptors employing neutralizing antibodies.

### Actin polymerization assay

The extent of actin polymerization was studied in the expanded cells. The expanded control and test HSPCs were exposed to 100 ng of SDF1α for different time points starting from 10–320 sec. After each time point, the cells were washed, fixed, permeabilized and transferred to a solution containing 1 U/ml fluorescein conjugated phalloidin and incubated for 30 minutes, followed by flow cytometry analysis.

### *In vivo* homing assay in NOD/SCID mice

After the expansion period, equal number (1×10^6^) of control and zVADfmk/zLLYfmk HSPCs were intra-venously infused to sub-lethally irradiated (300rads) NOD/SCID mice (5–6 weeks old). The study was conducted adhering to the institution's guidelines for animal husbandry and has been approved by the Institutional animal ethical committee named as National Centre for Cell Science Animal Ethical Committee(NCCS-AEC) – National Centre for Cell Science/Committee for Purpose of Control and Supervision of Experiments on Animals (NCCS –CPCSEA). Approval number of the study was: -Institutional Animal Care and Use Committee, Experimental Animal Facility - IACUC EAF/2004/B-71. Mice were housed in sterilized cages and received autoclaved food and water *ad libitum*. After 24 hours of transplantation, the animals were sacrificed and the spleen, tibia and femurs were collected. The cell suspensions from bone marrow, and spleen were blocked using mouse CD16/32 (mouse Fc blocker) and with human serum to eliminate non-specific binding. The cells were then stained using human specific CD45 and CD34 and acquired on FACS to assess the pan hematopoietic and donor progenitor cell homing respectively. Irradiated PBS (vehicle) - infused animals were kept to detect the background staining if any. In some experiments the expanded HSPCs were blocked using neutralizing antibodies for CXCR4, integrins and cell adhesion molecules prior to transplantation. The cells were analyzed by flow cytometry and a minimum of 100000 events acquired for each set.

### Statistical Analyses

The statistical differences between groups were analyzed by One-Way repeated measure analysis of variance using the software SIGMA STAT (Jandel Scientific Corporation, San Rafael, CA) for all the experiments. All comparisons were between control vs. zVADfmk and control vs. zLLYfmk. The values were plotted as mean ± standard deviation. Probability value: *p≤0.05, **p≤0.01, & ***p≤0.001 were considered statistically significant.

## Supporting Information

Figure S1 **Table summarizes the list of primer sequences used for qRT-PCR studies.**
(TIF)

Figure S2 **Higher number of primitive subset in the zVADfmk/zLLYfmk HSPCs. and the ‘priming’ effect of the inhibitors on immature CD34^+^ cells.** (A) Representative flow profile depicting a higher percentage of CD34^+^CXCR4^+^ subset in the zVADfmk/zLLYfmk HSPCs. (B) Three color flow cytometry analysis revealed a higher number of primitive CD34^+^CXCR4^+^CD38^−^ subset in the zVADfmk/ZLLYfmk expanded HSPCs. Representative flow cytometry profile showing CXCR4 vs. CD38 within the gated CD34 compartment (n = 3), (C) Over night treatment of isolated CD34^+^ cells with cytokines and zVADfmk/zLLYfmk resulted in an enhanced migration compared to the control. Data obtained from four experiments are represented as mean ± standard deviation, **p<0.01, n = 4. (D) Short-term treatment of CD34^+^ cells to zVADfmk/zLLYfmk did not alter the CD34^+^ population, but caused an improved migration of primitive CD34^+^CD38^−^ subsets compared to the control. A representative flow cytometry profile is shown (n = 4). (E) Representative flow cytometry profile showing an increase in the CXCR4^+^ population when the CD34^+^ cells were incubated with either zVADfmk or zLLYfmk compared to the control, n = 4.
(TIF)

Figure S3 **Higher expression of adhesion molecules on the total HSPCs.** (A) The zVADfmk/zLLYfmk HSPCs showed the presence of higher percentage of cells expressing CD62L on the surface. Data are represented as mean ± standard deviation of six experiments, *p<0.05. (B) The CD62L expression intensity found to be higher in the zVADfmk/zLLYfmk sets inferred from the immunostaining. Images were captured with confocal microscope. CD62L-green, nuclei-blue, bar = 10 µm. (C) Flow cytometry analysis showed a significant reduction in the CD62L cleaving enzyme TACE in the zVADfmk/zLLYfmk HSPCs compared to the control. (D) Expanded control and test HSPCs were immunostained against CD54 (ICAM-1) and CD44(HCAM) and images were captured by confocal microscopy. The presence of zVADfmk/zLLYfmk increased the expression of ICAM 1 and HCAM1 compared to the control, red- CD54 and green - CD44, blue – nuclei, bar = 10 µm.
(TIF)

Figure S4 **Higher expression of integrins CD49d and CD49e on total expanded HSPCs.** (A) Though the percentage of cells expressing the major integrins did not vary between control and inhibitor cultured HSPCs, the mean intensity of the integrins CD49d/VLA-4 and CD49e/VLA-5 were seen to be enhanced in the zVADfmk/zLLYfmk HSPCs. Data are represented as mean ± standard deviation of four experiments, *p<0.05, **p<0.01 (B) The expanded zVADfmk/zLLYfmk HSPCs showed a higher functional expression of β1 integrin epitope as assessed from HUTS 21 immunostaining after expansion. HUTS21-Green, nuclei-blue, scale bar 10 µm.
(TIF)

Figure S5 **Improved** ***in vivo*** **homing potential of zVADfmk/zLLYfmk HSPCs.** (A)Representative flow profile showing a higher number of human CD45 and huCD34 population within the bone marrow compartment of recipient mice (B) Representative flow profile showing a higher homing of human CD45^+^ cells to the spleen tissues of the recipient mice. However, the CD34^+^ progenitor cell homing to spleen was low and showed no marked difference between the control and test animals. (C) The human CFC recovery from the femurs and tibias of the NOD/SCID mice showed that the animals that received the zVADfmk/zLLYfmk HSPCs showed a higher CFC recovery in the presence of human growth factors confirming the observation of higher homing to bone marrow. Data are the mean ± standard deviation of three experiments, *p≤0.05.
(TIF)

Figure S6 **Effect of blocking of adhesion molecules on the** ***in vivo*** **homing of control and inhibitor-cultured HSPCs.** (A&B) The expanded HSPCs were blocked for CD44, CD62L, and CD54 prior to transplantation and the human cell homing was detected after 24 hours using huCD45 marker. There was no change in the homing efficiency of unblocked vs adhesion molecules blocked sets in the BM homing. A moderate reduction in spleen homing was observed (Figure S6B) but was not significant when compared to the respective unblocked sets, n = 3 independent experiments.
(TIF)
