# Supplementary figures and images for: Pharmacological Inhibition of Caspase and Calpain Proteases: A Novel Strategy to Enhance the Homing Responses of Cord Blood HSPCs during Expansion
Source: PLoS One. 2012 Jan 3;7(1):e29383. doi: 10.1371/journal.pone.0029383 (PMC3250442; doi:10.1371/journal.pone.0029383)

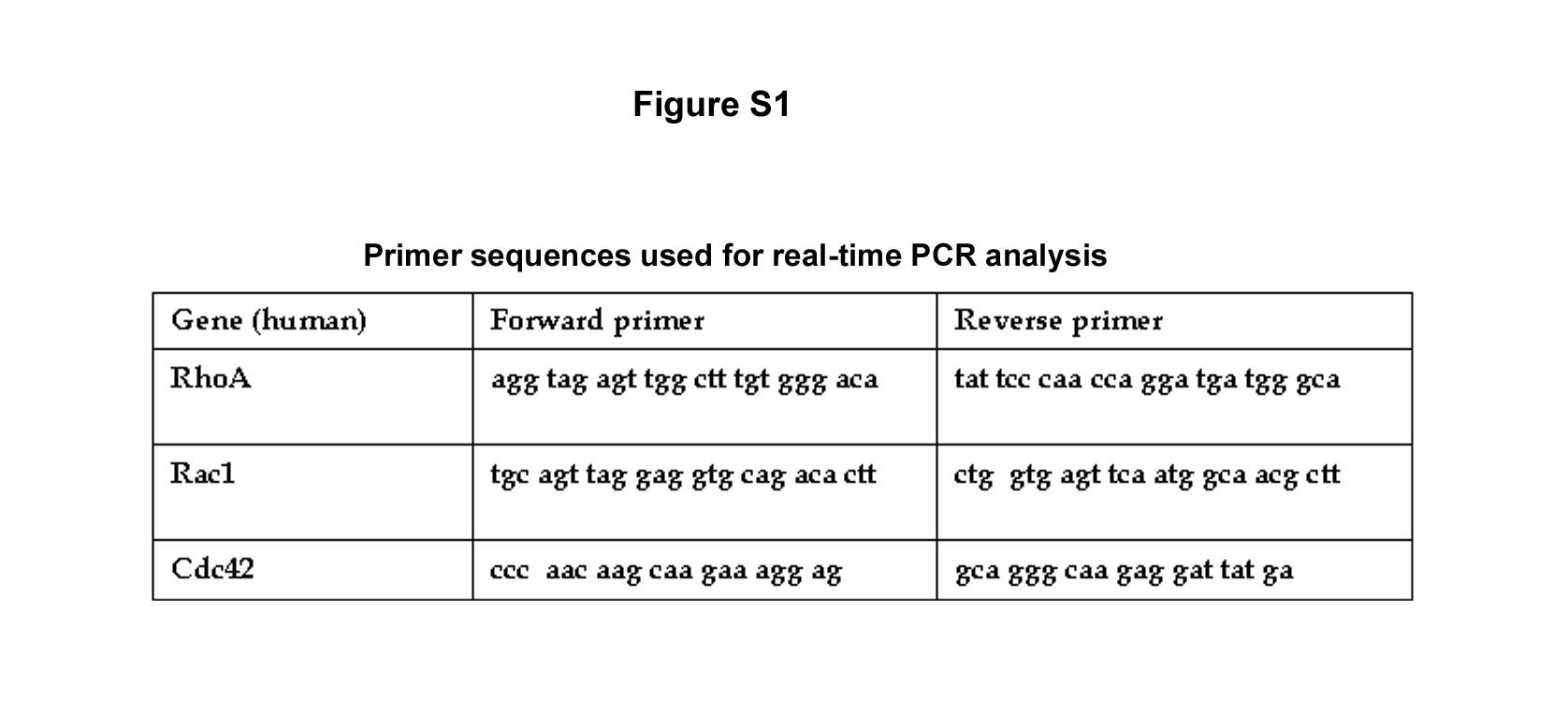

Supplement: Figure S1 — Table summarizes the list of primer sequences used for qRT-PCR studies. (TIF) [file pone.0029383.s001.tif]

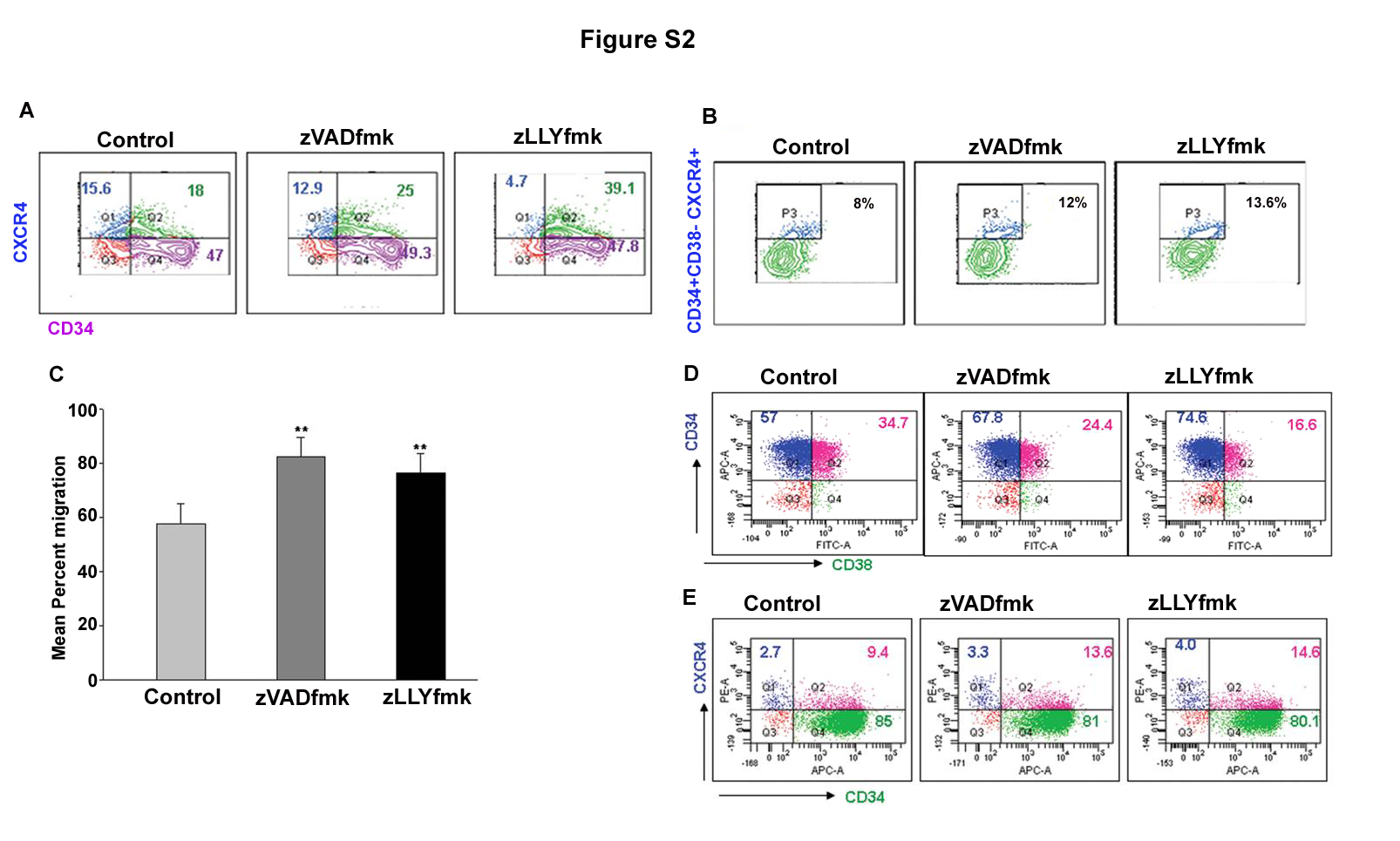

Supplement: Figure S2 — Higher number of primitive subset in the zVADfmk/zLLYfmk HSPCs. and the ‘priming’ effect of the inhibitors on immature CD34+ cells. (A) Representative flow profile depicting a higher percentage of CD34+CXCR4+ subset in the zVADfmk/zLLYfmk HSPCs. (B) Three color flow cytometry analysis revealed a higher number of primitive CD34+CXCR4+CD38− subset in the zVADfmk/ZLLYfmk expanded HSPCs. Representative flow cytometry profile showing CXCR4 vs. CD38 within the gated CD34 compartment (n = 3), (C) Over night treatment of isolated CD34+ cells with cytokines and zVADfmk/zLLYfmk resulted in an enhanced migration compared to the control. Data obtained from four experiments are represented as mean ± standard deviation, **p<0.01, n = 4. (D) Short-term treatment of CD34+ cells to zVADfmk/zLLYfmk did not alter the CD34+ population, but caused an improved migration of primitive CD34+CD38− subsets compared to the control. A representative flow cytometry profile is shown (n = 4). (E) Representative flow cytometry profile showing an increase in the CXCR4+ population when the CD34+ cells were incubated with either zVADfmk or zLLYfmk compared to the control, n = 4. (TIF) [file pone.0029383.s002.tif]

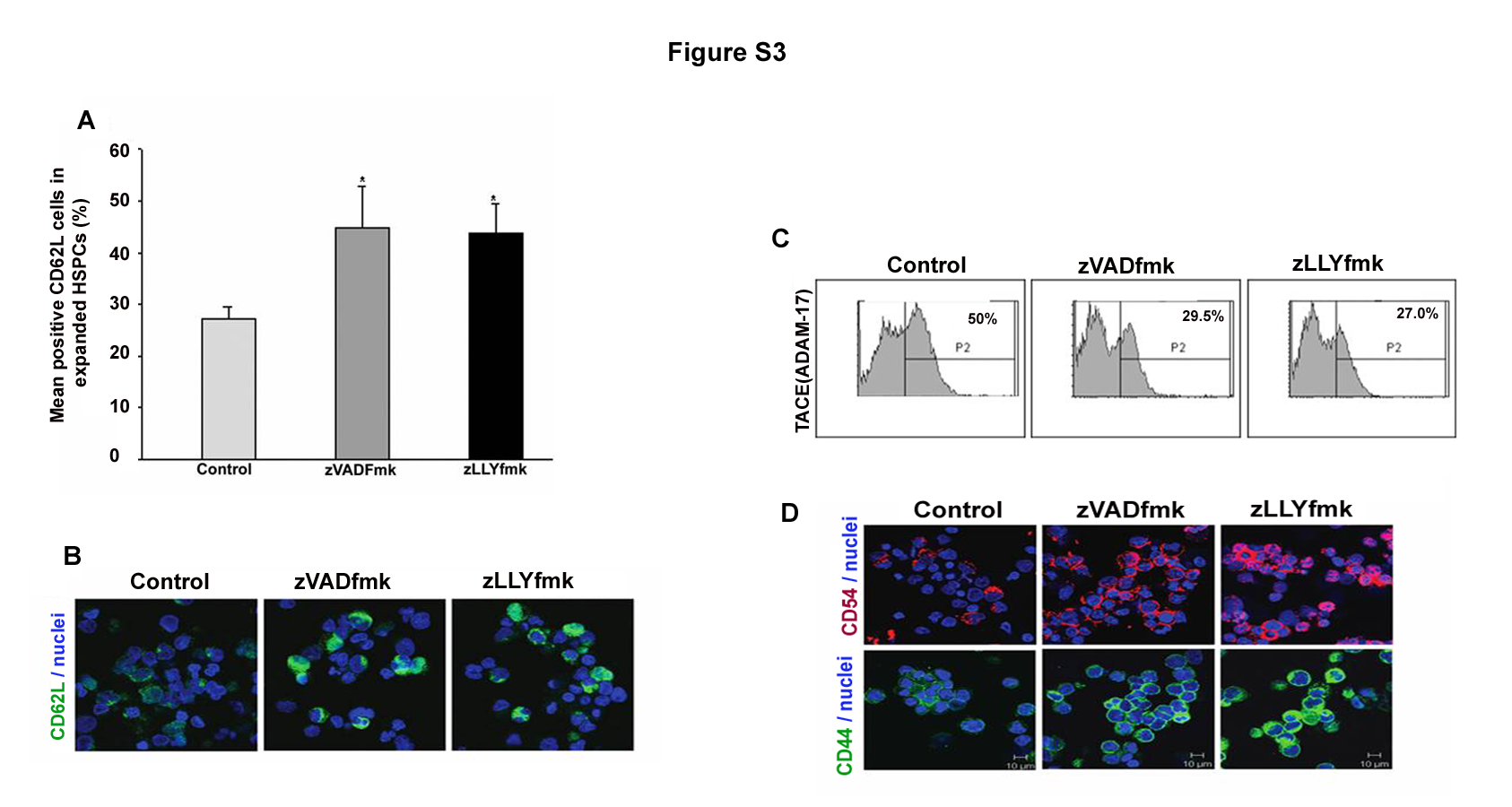

Supplement: Figure S3 — Higher expression of adhesion molecules on the total HSPCs. (A) The zVADfmk/zLLYfmk HSPCs showed the presence of higher percentage of cells expressing CD62L on the surface. Data are represented as mean ± standard deviation of six experiments, *p<0.05. (B) The CD62L expression intensity found to be higher in the zVADfmk/zLLYfmk sets inferred from the immunostaining. Images were captured with confocal microscope. CD62L-green, nuclei-blue, bar = 10 µm. (C) Flow cytometry analysis showed a significant reduction in the CD62L cleaving enzyme TACE in the zVADfmk/zLLYfmk HSPCs compared to the control. (D) Expanded control and test HSPCs were immunostained against CD54 (ICAM-1) and CD44(HCAM) and images were captured by confocal microscopy. The presence of zVADfmk/zLLYfmk increased the expression of ICAM 1 and HCAM1 compared to the control, red- CD54 and green - CD44, blue – nuclei, bar = 10 µm. (TIF) [file pone.0029383.s003.tif]

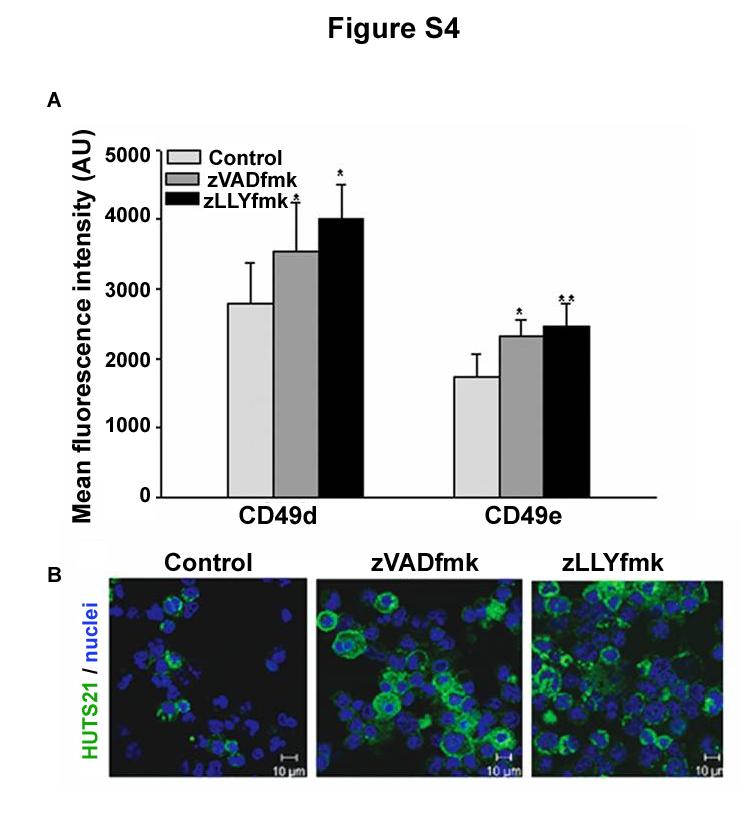

Supplement: Figure S4 — Higher expression of integrins CD49d and CD49e on total expanded HSPCs. (A) Though the percentage of cells expressing the major integrins did not vary between control and inhibitor cultured HSPCs, the mean intensity of the integrins CD49d/VLA-4 and CD49e/VLA-5 were seen to be enhanced in the zVADfmk/zLLYfmk HSPCs. Data are represented as mean ± standard deviation of four experiments, *p<0.05, **p<0.01 (B) The expanded zVADfmk/zLLYfmk HSPCs showed a higher functional expression of β1 integrin epitope as assessed from HUTS 21 immunostaining after expansion. HUTS21-Green, nuclei-blue, scale bar 10 µm. (TIF) [file pone.0029383.s004.tif]

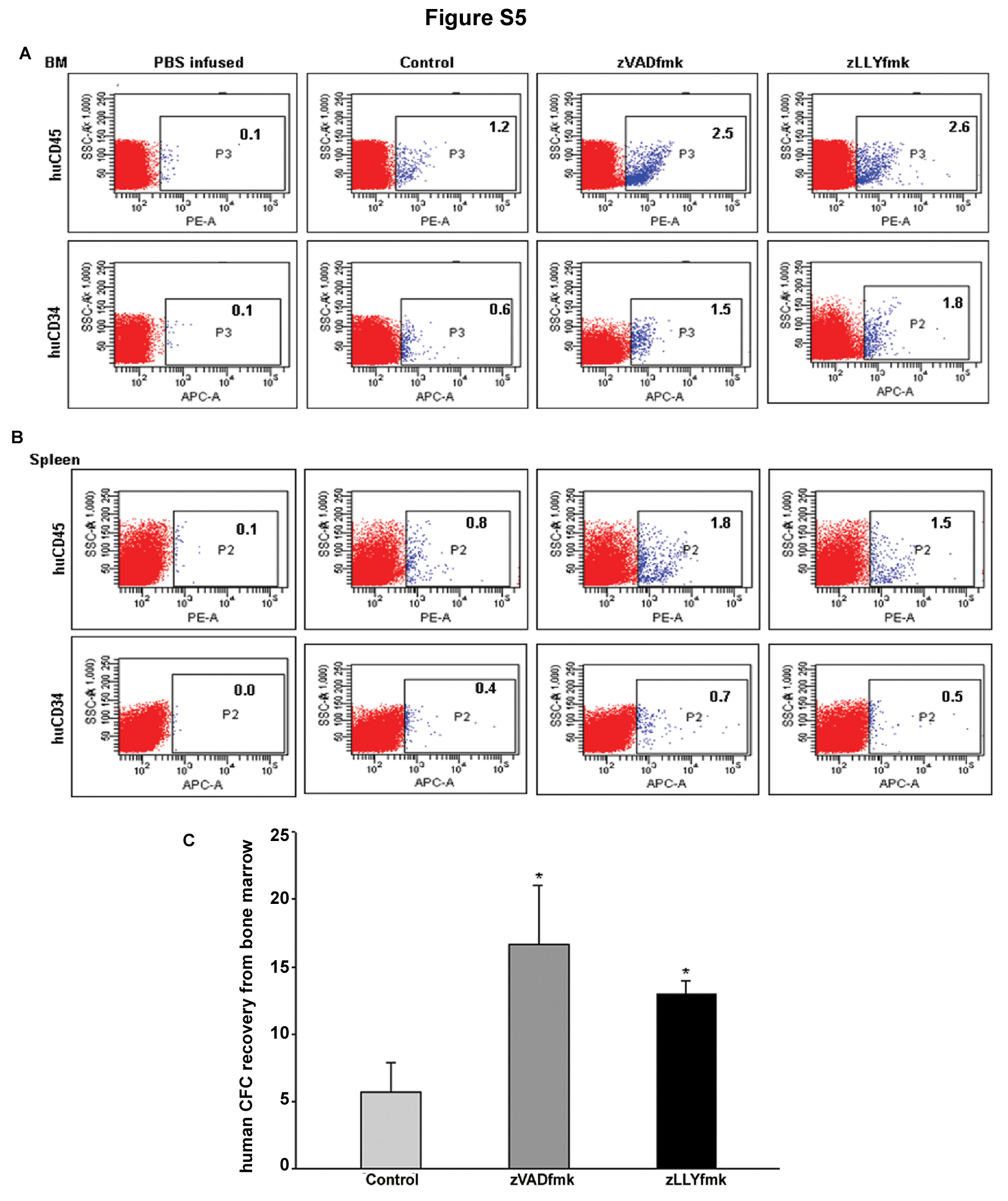

Supplement: Figure S5 — Improved in vivo homing potential of zVADfmk/zLLYfmk HSPCs. (A)Representative flow profile showing a higher number of human CD45 and huCD34 population within the bone marrow compartment of recipient mice (B) Representative flow profile showing a higher homing of human CD45+ cells to the spleen tissues of the recipient mice. However, the CD34+ progenitor cell homing to spleen was low and showed no marked difference between the control and test animals. (C) The human CFC recovery from the femurs and tibias of the NOD/SCID mice showed that the animals that received the zVADfmk/zLLYfmk HSPCs showed a higher CFC recovery in the presence of human growth factors confirming the observation of higher homing to bone marrow. Data are the mean ± standard deviation of three experiments, *p≤0.05. (TIF) [file pone.0029383.s005.tif]

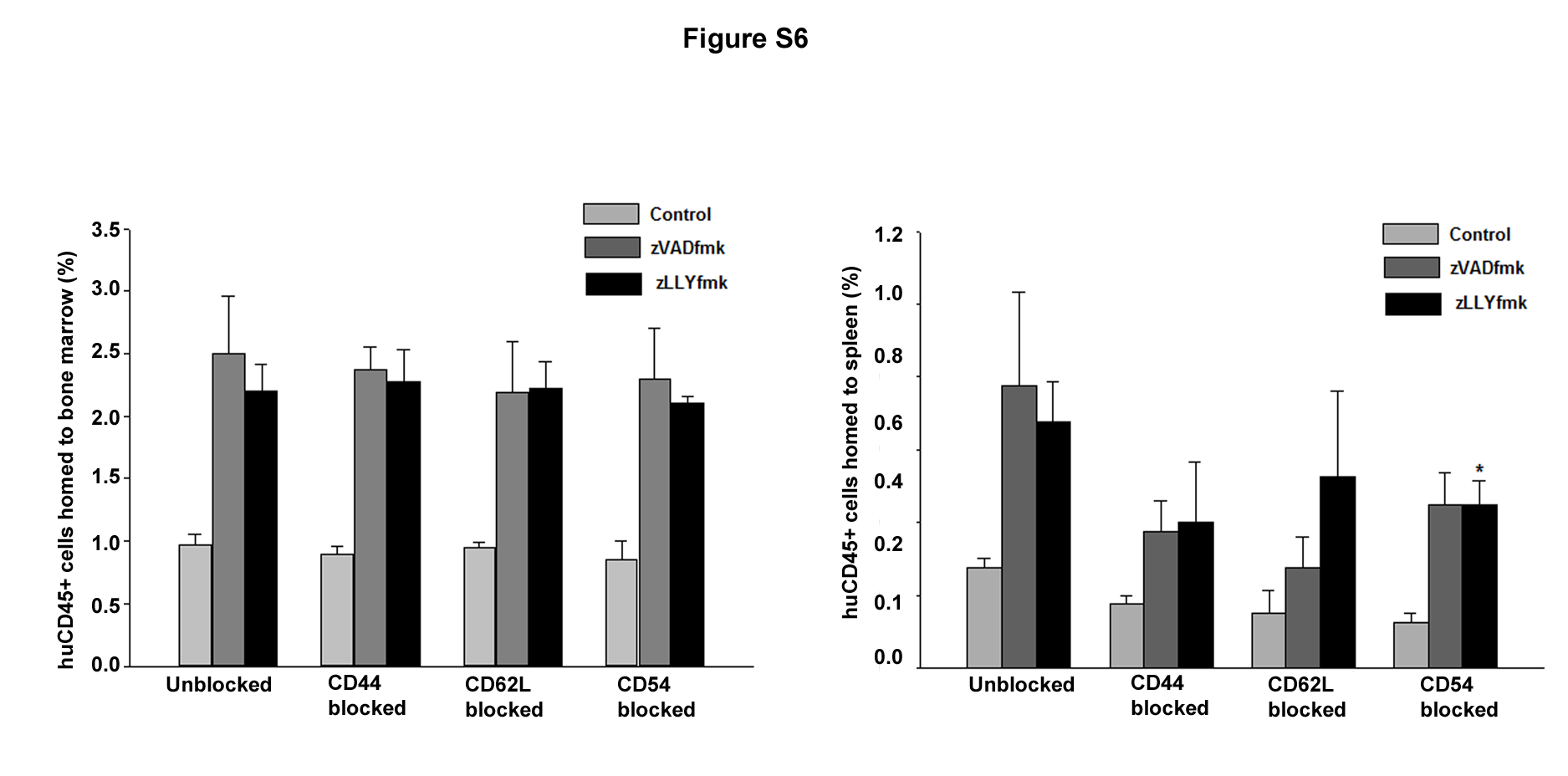

Supplement: Figure S6 — Effect of blocking of adhesion molecules on the in vivo homing of control and inhibitor-cultured HSPCs. (A&B) The expanded HSPCs were blocked for CD44, CD62L, and CD54 prior to transplantation and the human cell homing was detected after 24 hours using huCD45 marker. There was no change in the homing efficiency of unblocked vs adhesion molecules blocked sets in the BM homing. A moderate reduction in spleen homing was observed (Figure S6B) but was not significant when compared to the respective unblocked sets, n = 3 independent experiments. (TIF) [file pone.0029383.s006.tif]
